# Quality Control of the Root and Rhizome of *Helminthostachys zeylanica* (*Daodi-Ugon*) by HPLC Using Quercetin and Ugonins as Markers

**DOI:** 10.3390/molecules22071115

**Published:** 2017-07-05

**Authors:** Kun-Chang Wu, Chun-Pin Kao, Yu-Ling Ho, Yuan-Shiun Chang

**Affiliations:** 1 Department of Chinese Pharmaceutical Sciences and Chinese Medicine Resources, College of Chinese Medicine, China Medical University, Taichung 40402, Taiwan; johnwu0919@gmail.com; 2 Department of Nursing, Hsin Sheng Junior College of Medical Care and Management, Taoyuan 32544, Taiwan; chunpin@hsc.edu.tw; 3 Department of Nursing, Hungkuang University, Taichung 43302, Taiwan; 4 Chinese Crude Drug Pharmacy, China Medical University Hospital, Taichung 40402, Taiwan

**Keywords:** quality control, *Helminthostachys zeylanica*, *Daodi-Ugon*, Quercetin, Ugonin J, Ugonin M

## Abstract

*Daodi-Ugon* is the dried root and rhizome of *Helminthostachys zeylanica* (L.) Hook. and has been used for centuries in the treatment of inflammation, fever, pneumonia, burns, and various disorders. However, the chromatographic methods to determine the phytochemical composition of *H. zeylanica* have never been reported. This study not only aims to develop a valid high-performance liquid chromatography (HPLC) method and to establish a chromatographic fingerprint for the quality control of *H. zeylanica*, it also establish the proposed content limits of Quercetin, Ugonin J, and Ugonin M. An HPLC method with a RP_18_ column (250 × 4.6 mm, 5 μm) was developed for the quantitative analysis of Quercetin, Ugonin J, and Ugonin M in *H. zeylanica*. A simple gradient of (A) methanol/(B) phosphoric acid in water (5–45 min, 70–80% A; 50–55 min, 80–70% A) was used and 360 nm was selected as the detection wavelength. The average contents and proposed content limits for *H. zeylanica* were calculated with a *t*-test and a measurement uncertainty test based on 20 batches of authentic *H. zeylanica* samples. Limits of detection (LOD), quantification (LOQ), linearity, precision, repeatability, stability, and recovery of the developed method were validated. All of the validation results of quantitative determination and fingerprinting methods were satisfactory. The developed method was then applied to assay the contents of Quercetin, Ugonin J, and Ugonin M and to acquire the fingerprints of all of the collected *H. zeylanica* samples. At the 99% confidence level, the calculated content limits were 56.45, 112.15, and 277.98 mg/kg for Quercetin, Ugonin J, and Ugonin M, respectively. Those validated HPLC quantitative method, fingerprinting profile, and the proposed content limits of three chemical markers that could be used in the quality control of *H. zeylanica* in the market.

## 1. Introduction

Herbal medicine has gained popularity in many countries throughout the years. With the increased trend of usage worldwide, the assessment of safety, quality, and efficacy of these medicines has been an important concern for health professionals and healthcare authorities [1].

**Figure 1:**
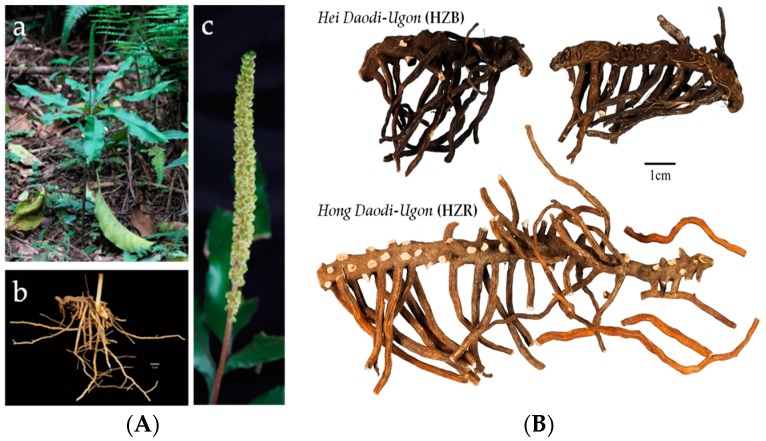
(**A**) The original plant of HZ: (**a**) fronds tripartite, each division with two to three (or more) lanceolate lobes; (**b**) root and rhizome creeping, fleshy and fronds usually single at rhizome apex; (**c**) spikelike sporophore arising at top of common stipe; (**B**) The herbal HZ samples of *Hei Daodi-Ugon*: HZB and *Hong Daodi-Ugon*: HZR.

The root and rhizome of *Helminthostachys zeylanica* (L.) Hook. (Family: Ophioglossaceae), which is known as *Daodi-Ugon* in Chinese, has been used for centuries in the treatment of inflammation, fever, pneumonia, burns, and various disorders [2]. From our investigation, we found that the surface of herbal *H. zeylanica* (HZ) samples appeared slightly black or slightly red (Figure 1), with the commercial names *Hei Daodi-Ugon* (HZB) and *Hong Daodi-Ugon* (HZR) respectively, and some herbal providers claim HZR is superior to HZB in both quality and efficacy.

Recently, several chemical components of HZ—those containing stilbenes and flavonoids such as Quercetin, Ugonin J, and Ugonin M—have been published [3,4]. Many Ugonins have been discovered and most of them have only been evaluated through in vitro experiments. They were found to exhibit the antioxidant activities [4,5], anti-inflammation [4,6,7,8,9], melanogenesis inhibitory activities [10,11]; and neuroprotective [12], antiosteoporosis [9,13,14], anti-cancer [15], and immunomodulatory effects [16]. Moreover, some researchers have used the crude extract of HZ to determine anti-inflammatory effects, such as hepatotoxicity [17,18] and acute lung injury [19] models, in mice.

To figure out the possible active ingredient of anti-inflammation of HZ, we have focused on isolating the pure compounds from HZ. Based on our previous work [20], we have preliminarily determined Ugonin M, the major compound of HZ, as the active constituent of antioxidant and anti-inflammation of HZ via in vivo experiment. Another compound in HZ which is Ugonin J also exhibited the anti-oxidant [5] and anti-inflammatory activities [9] (Supplementary File 1). As for Quercetin, another compound in HZ, previous pharmacological studies have demonstrated that it has an antioxidant activity, anti-inflammatory effects, hepatoprotective activities, etc. [21,22,23,24]. Moreover, Ugonin J and Ugonin M are two unique flavonoids, and they have only been isolated from HZ [6]. In this study, Quercetin, Ugonin J, and Ugonin M are used as marker compounds for quality evaluation of HZ.

A combination of fingerprint analysis and quantitative analysis are the main method for controlling the efficacy and quality of herbal medicines and has been accepted by the World Health Organization (WHO) [25,26,27,28,29]. Many researchers have given their best effort in researching the pharmacological activity of HZ. However, a validated HPLC quantitative method and fingerprinting profile for HZ involving unique markers to ensure the quality and efficacy as well as avoid adulteration have not been reported so far. Therefore, strengthening the quality control and the regulation of this herb is therefore important to safeguard patients’ interests, safety, and the efficacy of this herb. On the other hand, the clarification of quality between HZB and HZR by using statistical model is also a novelty of this study.

This study aimed to develop a simple and reliable analytical method for the quality control of HZ, as well as to preliminarily determine Quercetin, Ugonin J, and Ugonin M as the active constituents of anti-inflammation of HZ. Based on the developed method, 20 batches of HZ were investigated and their content limits were statistically calculated, and their characteristic peaks were determined via their overlay chromatograms. The results should be able to identify their authenticity and provide the proposed content limits of Quercetin, Ugonin J, and Ugonin M as content standards in the quality control of HZ.

## 2. Results and Discussion

### 2.1. Optimization of the Extraction Method

**Figure 2:**
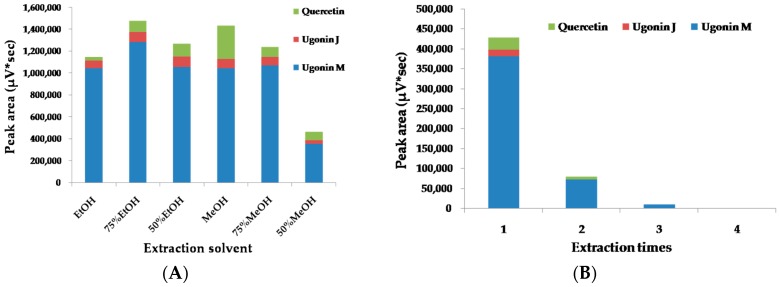
Influence of (**A**) extraction solvent and (**B**) number of extraction times on the extraction efficiency of three analytes in HZ.

The extraction solvents were optimized by taking the extraction efficiency of Quercetin, Ugonin J, and Ugonin M as indexes. HZ powder (1.0 g) was extracted for 30 min with different solvents (15 mL). The results showed that the total contents of the three analytes obtained with 75% ethanol were the highest as compared to the values obtained from other solvents (Figure 2A). Moreover, the three analytes were almost extracted completely (>99%) on the third time (Figure 2B). Thus, the optimal extraction procedure as described in the “3.3. Sample and Reference Preparation” section was established.

### 2.2. Optimization of Chromatographic Conditions

To develop a reliable chromatographic fingerprinting method, an optimized strategy for HPLC conditions was performed. To obtain sharp and symmetrical peaks, different mobile phase systems, including methanol–water and acetonitrile–water elution systems, were tested. The results demonstrated a good resolution and baseline and sharp and symmetrical peaks were obtained by using a methanol–phosphoric acid in water (0.1%, *v*/*v*) system, similar to a method used in a previous report [20]. A few different columns: Waters XTerra RP18 (Milford, MA, USA), Merck Purospher^®^ STAR RP18 (Billerica, MA, USA), and Grace Alltima C18 (Deerfield, IL, USA) were tested before the Merck Purospher^®^ STAR RP18 column (250 mm × 4.6 mm i.d., 5 μm) was finally selected. To obtain a sufficiently large number of detectable peaks in the chromatographic fingerprints, a PAD full scan (190–400 nm) was used to acquire all the main peaks, with 360 nm finally being selected as the detection wavelength. Gradient time, gradient procedure, and initial composition of the mobile phase were all taken into consideration in the process of gradient optimization. Finally, the gradient procedure was optimized as described in the “3.4. HPLC Analysis” section.

### 2.3. Validation of the Quantitative Analytical Method

Method validation parameters included LOD, LOQ, linearity, precision, repeatability, stability, and recovery.

The calibration curves were plotted on the basis of linear regression analysis of the integrated peak areas (*y*) versus concentrations (*x*, mg/L) of the three analytes at five different levels. LOD and LOQ values for each analyte under the present chromatographic conditions were determined in terms of baseline noise, according to the IUPAC definition. LOD was determined as the analyte concentration yielding signal with a single-to-noise (S/N) ratio of 3:1, whereas the LOQ was defined as the analyte concentration yielding signal with S/N ratio at 10:1. The results of regression equations, correlation coefficients, linear ranges, LODs, and LOQs for Quercetin, Ugonin J, and Ugonin M are shown in Table 1. The correlation coefficient (*R*^2^) of the regression equation for each analyte indicates good linearity, being better than 0.9999.

For the precision test, one of the mixed standard solutions (Quercetin, 1.56 μg/mL; Ugonin J, 3.13 μg/mL; Ugonin M, 25 μg/mL) was analyzed with five successive injections. The RSDs for the peak areas were calculated as measurements of precision. The RSDs of precision variation for Quercetin, Ugonin J, and Ugonin M were less than 1.5%, as shown in Table 1. Repeatability was evaluated by analyzing five different working solutions prepared from the same sample (HZR-01). RSD values were 1.80, 4.84, and 1.89% for Quercetin, Ugonin J, and Ugonin M, respectively (Table 1). Stability was determined using repeated analyses of the same sample solution at different times (0, 2, 4, 8, 16, and 24 h) during storage at room temperature (approx. 30 °C) over a 24 h period. The RSD values of peak areas of Quercetin, Ugonin J, and Ugonin M were 3.10, 3.89, and 2.57%, respectively (Table 1), indicating that the stability of the sample solutions within one day was satisfactory. Recovery tests were determined using spiked HZR-01 samples. A portion of 0.5000 g of a HZR-01 sample was spiked with 0.0040 mg of Quercetin, 0.0700 mg of Ugonin J, and 0.6400 mg of Ugonin M, respectively. Five replicate samples were extracted and analyzed according to the procedures described above. As shown in Table 1, the mean recoveries (*n* = 5) are 94.37 ± 3.32%, 95.98 ± 4.77%, and 97.53 ± 1.26%, respectively. These RSD values indicate that the proposed methodology is reproducible and suitable for the quantitative determination of Quercetin, Ugonin J, and Ugonin M in HZ samples.

### 2.4. Quantitative Determination of the Three Analytes in HZ Samples

**Figure 3:**
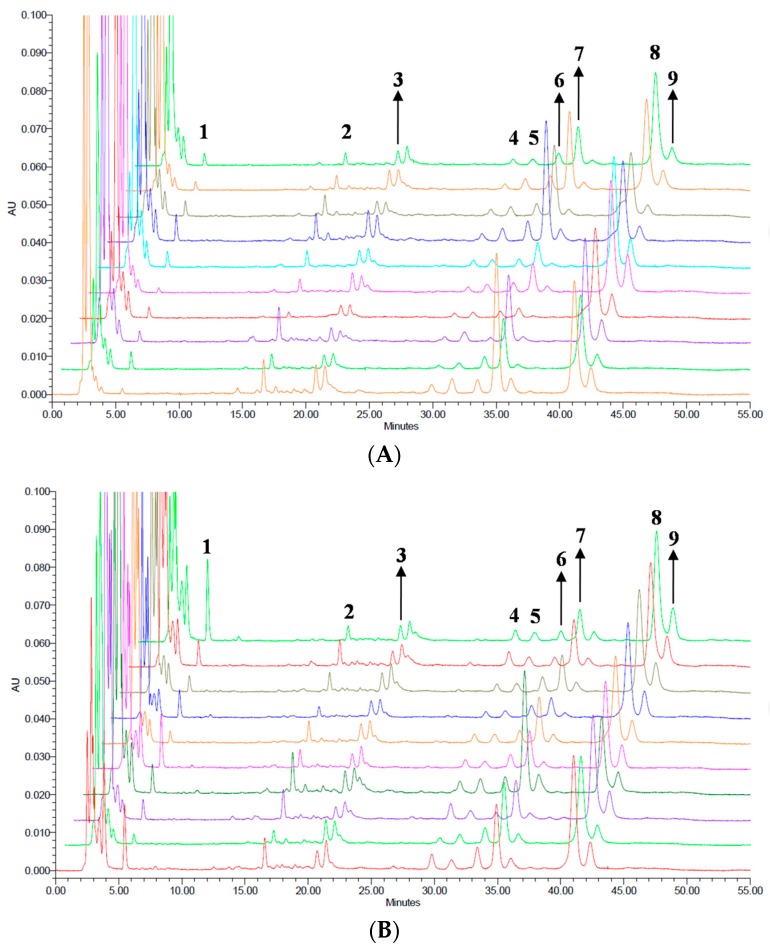
HPLC chromatogram fingerprint profiles of (**A**) 10 batches of HZR and (**B**) 10 batches of HZB. The number 1–9 indicate the common fingerprinting peaks for identification. Peak 1: Quercetin, peak 4: Ugonin J, and peak 7: Ugonin M.

The developed HPLC-PAD analytical method was applied to the quantitative determination of Quercetin, Ugonin J, and Ugonin M in 20 batches of HZ samples. The fingerprint technique is considered an effective method for the quality of Traditional Chinese medicines (TCMs) because it describes all the characteristics ingredients of the TCMs [25,30]. The integrated feature of overlay chromatograms for 20 batches of HZ were shown in Figure 3. The chromatographic patterns of all HZR (Figure 3A) and HZB (Figure 3B) samples were highly consistent, although the abundance of some peaks obviously varied. With the proposed chromatographic methods, there were a total of nine characteristic peaks in the chromatogram and those peaks could be used as the fingerprinting features for HZ authentication.

Although the content of Quercetin is comparatively low in HZ, it exhibited an array of pharmacological activities [21]. In this study, peak 1 (Quercetin) was chosen as the marker peak. The relative retention times (RRTs) and the relative standard deviation of retention times (RSDs) of the nine characteristic peaks of HZ are listed in Table 2. For positive identification, it is suggested that the sample must have shown the above nine characteristic peaks with RRTs falling within the acceptable range of the corresponding peaks in the fingerprint chromatogram.

The calibration curves were used to calculate the contents of the three compounds in HZ samples (data are shown in Table 3). To begin with, the contents of Quercetin, Ugonin J, and Ugonin M in different samples varied from 0.0378 to 0.4693 mg/g, 0.0639 to 0.4177 mg/g, and 0.1016 to 1.4685 mg/g, respectively. The highest total content of Quercetin, Ugonin J, and Ugonin M was found in HZB-04 at 1.7633 mg/g; conversely, HZR-04 had the lowest content at 0.2690 mg/g. Based on the results in Table 3, HZR and HZB samples did not differ significantly from each other in the total content of Quercetin, Ugonin J, and Ugonin M when compared by one-way ANOVA (Table 4). These findings demonstrated that HZR is not superior to HZB and both HZR and HZB can produce equivalent quality.

As is well known, geography, climate, cultivation, harvesting, storage, and post-harvest treatment may cause fluctuations in herbal constituents [30,31,32]. Nevertheless, in this study, we found that the distribution of herbal secondary metabolites in the herbal tissue to be consistent. Therefore, we estimated the content ranges of Quercetin, Ugonin J, and Ugonin M for the HZ population using data from the collected representative samples via statistical inference (Supplementary File 2) [33,34,35]. At the 99% confidence level, the calculated content limits were 56.45, 112.15, and 277.98 mg/kg for Quercetin, Ugonin J, and Ugonin M, respectively (Table 5). These content limits can be used as reference values to evaluate the quality of HZ samples in future.

## 3. Materials and Methods

### 3.1. Herbal Materials

To determine the difference between HZR and HZB in phytochemical composition, we respectively collected 10 batches of HZB and HZR from herbal pharmacies in Taiwan, and marked them as HZR-01 to HZR-10 and HZB-01 to HZB-10 according to their differences in macroscopic appearances (Table 3). All the herbal samples were authenticated by professor Yuan-Shiun Chang (China Medical University), and the specimens were deposited in the Department of Chinese Pharmaceutical Sciences and Chinese Medicine Resources in the College of Chinese Medicine at China Medical University.

### 3.2. Reagents

The reference compound of Quercetin used in this study was purchased from the Shanghai R & D Center for Standardization of Traditional Chinese Medicine (Shanghai, China), while the Ugonin J and Ugonin M were isolated from HZ in our laboratory (purity: higher than 98%, the ^1^D-NMR spectroscopic data are shown in Supplementary File 3) [20]. LC-grade methanol was purchased from the Merck Co. branch in Taipei, Taiwan. Purified water was prepared with a Milli-Q system (Millipore, Milford, MA, USA). All other reagents used in the present study were of analytical grade.

### 3.3. Sample and Reference Preparation

The sample and reference preparation were investigated by different extraction times and extraction solvents: ethanol, 75% ethanol, 50% ethanol, methanol, 75% methanol, and 50% methanol all with ultrasonic extraction at room temperature. Considering the extraction efficiency and relative study [27,28,30], we have determined the sample and reference preparation method and conducted subsequent HPLC experiments by the following procedures: all HZ samples were ground into a fine powder (20 mesh) using a grinder with a knife blade. Each HZ powder sample (1.0 g) was carefully weighed into a 50 mL centrifuge tube. 75% ethanol (15 mL) was then added to the tube and shaken gently. Each sample was then extracted with an ultrasonic cleaner (Delta DC400H, Taiwan Delta New Instrument Co., Taipei, Taiwan) at a frequency of 40 kHz at 25 °C for 30 min. The extract of each sample was centrifuged for 10 min at 3000 rpm, and the supernatant was then transferred to a 50 mL volumetric flask. The procedure was repeated a total of three times, and the supernatants were combined. The final volume was made up to 50 mL with 75% ethanol which was then filtered through a 0.45 μm PVDF syringer filter (VWR Scientific, Seattle, WA, USA) before analysis. The reference compounds of Quercetin, Ugonin J, and Ugonin M were accurately weighed respectively and were dissolved in 75% ethanol as stock solutions. The stock solutions were then diluted to their appropriate concentrations for the establishment of calibration curves. An aliquot of 10 μL of each solution was used for HPLC analyses.

### 3.4. HPLC Analysis

HPLC analyses were performed on a Waters 2695 HPLC system equipped with a Waters 2998 photodiode array detector (PAD, Waters Corporation, Milford, MA, USA), a Waters e2695 separations module and a column heater module. A Merck Purospher^®^ STAR Shield RP 18 column (250 × 4.6 mm, 5 μm, EMD Millipore Corporation, Billerica, MA, USA) was used. The reliable chromatographic fingerprinting method was reported in our previous study [20] *viz* the mobile phase consisted of (A) methanol and (B) phosphoric acid in water (0.1%, *v*/*v*). The optimized elution conditions were as follow: 5–45 min, 70–80% A; 50–55 min, 80–70% A. The flow rate was 1 mL/min, and the injection volume was 10 μL. UV spectra were acquired from 190 nm to 400 nm. The autosampler and the column compartment were maintained at 25 °C and 35 °C, respectively.

### 3.5. Data Analysis

The proposed content limit for each analyte was calculated according to the contents from 20 batches with consideration of the standard uncertainty of the precision, bias, purity of reference substance, and water content (the calculation equation is shown in Supplementary File 2). The calculation was based on the ISO “Guide to the Expression of Uncertainty in Measurement” [34], the EURACHEM/CITAC document “Quantifying Uncertainty in Analytical Measurement” [33], and the LGC document “Development and Harmonization of Measurement Uncertainty Principles” [35]. The mean differences for three analytes between HZR and HZB were evaluated by the analysis of variance (ANOVA).

## 4. Conclusions

The present work is the first report to develop a valid HPLC method with three reference chemical markers to evaluate the quality of commercial HZ herbal materials. The nine characteristic peaks in fingerprinting chromatogram recorded could also be used for HZ authentication, and the proposed content limits of the three analytes could be used as reference values to determine the quality of HZ herbal material used in pharmaceutical manufacturing factories. There is no difference in the composition of HZR and HZB in terms of the total content of the three analytes, Quercetin, Ugonin J, and Ugonin M. Concisely, HZ quality is consistent regardless of its external color.

## Figures and Tables

**Table 1:** Linearity, precision, repeatability, recovery, LOD, and LOQ of the established method.

Parameter	Quercetin	Ugonin J	Ugonin M
Regression data			
Regression quation ^a^	*y* = 29,781 *x* − 6491	*y* = 12,619 *x* − 3005	*y* = 36,605 *x* − 11,200
*R*^2^	*R*^2^ = 1.0000	*R*^2^ = 0.9999	*R*^2^ = 1.0000
LinearityRange (μg/mL)	0.58–9.25	1.56–50.00	1.56–50.00
LOD ^b^ (μg/mL)	0.29	0.78	0.39
LOQ ^c^ (μg/mL)	0.58	1.56	0.78
Precision (RSD, %)	0.16	1.32	0.36
Repeatability (RSD, %)	1.80	4.84	1.89
Stability (RSD, %)	3.10	3.89	2.57
Mean recovery (%)	94.37	95.98	97.53
	91.05–97.69	91.21–100.75	96.27–98.79

^a^ *y* is the peak area in UV chromatograms detected in 360 nm, *x* is the concentration (μg/mL) of the analyte; ^b^ LOD refers to the limit of detection, S/N = 3; ^c^ LOQ refers to the limit of detection, S/N = 10.

**Table 2:** The RRTs ^a^ and RSDs of the nine characteristic peaks.

Peaks No.	RRT	RSD (%)
1 (Quercetin, marker)	1.00	0
2	3.02	0.15
3	3.77	0.15
4 (Ugonin J)	5.42	0.22
5	5.70	0.27
6	6.08	0.20
7 (Ugonin M)	6.35	0.17
8	7.46	0.20
9	7.70	0.19

^a^ RRT = retention time of the characteristic peak/retention time of the Quercetin.

**Table 3:** Contents of three analytes in different HZ samples.

Sample No.	Content (mg/g Dry Weight)			Moisture Content (%)
	Quercetin ^a^	Ugonin J ^a^	Ugonin M ^a^	
HZR-01	0.0417	0.2134	1.4685	12.08
HZR-02	0.1075	0.0639	0.5180	10.74
HZR-03	0.0728	0.0973	0.7065	12.32
HZR-04	0.0713	0.0961	0.1016	11.62
HZR-05	0.0378	0.1152	0.3048	11.65
HZR-06	0.1024	0.1574	0.2402	11.67
HZR-07	0.1557	0.1790	1.2591	11.38
HZR-08	0.0922	0.1552	0.7425	11.82
HZR-09	0.0610	0.1358	0.8184	12.60
HZR-10	0.0816	0.1264	0.3987	11.45
HZB-01	0.3669	0.3889	0.6915	10.71
HZB-02	0.0665	0.1356	0.6256	11.22
HZB-03	0.1236	0.4177	0.4005	12.59
HZB-04	0.1679	0.3217	1.2737	11.83
HZB-05	0.3087	0.1967	0.3759	10.77
HZB-06	0.0765	0.1985	0.4723	10.39
HZB-07	0.1661	0.1490	0.1967	12.11
HZB-08	0.1018	0.1885	0.3758	11.45
HZB-09	0.1554	0.3420	0.5075	11.66
HZB-10	0.4693	0.2613	0.3243	11.36

^a^ The content was calculated on dry weight basis.

**Table 4:** Comparison of HZR and HZB in terms of total content of Quercetin, Ugonin J, and Ugonin M using ANOVA test.

Source of Variation	SS ^a^	Df ^b^	MS ^c^	F ^d^	F Crit ^e^
Between Groups	0.063	1	0.063	0.343	0.565
Within Groups	3.309	18	0.184		
Total	3.372	19			

^a^ Sum of square ; ^b^ Degree of freedom; ^c^ Mean square; ^d^ Calculated F value; ^e^ Critical F at 95% confidence interval.

**Table 5:** Mean contents and proposed content limits for three analytes in HZ.

Parameters	Quercetin	Ugonin J	Ugonin M
Ca ^a^	140.86	196.72	550.45
SD ^b^	113.34	98.88	352.65
RSD ^c^	80.47	50.26	64.07
N ^d^	20	20	20
t_critical_^e^	2.539	2.539	2.539
*uc* (C) ^f^	0.0712	0.0723	0.0656
Calculated limit (mg/kg) ^g^	56.45	112.15	277.98
Proposed limit (%) ^h^	0.0055	0.011	0.025
Outlier ^i^	0	0	0
Failing rate (%) ^j^	10	15	15

^a^ Ca, average content of three analytes in samples (mg/kg); ^b^ SD, standard deviation of the contents in all samples; ^c^ RSD (%) = 100 × SD/mean; ^d^ n, sample size; ^e^ t_critical_, critical value for a 99% confidence interval when the sample size was 20; ^f^ *uc* (C), combined standard uncertainty of the three analytes content in the sample (mg/kg). The calculation is shown in the Supplementary File 2; ^g^ Calculated limit for three analytes in the samples. The calculation is shown in the Supplementary File 2; ^h^ a suggestive specification of content of chemical marker not less than proposed limit (%); ^i^ Number of outlier samples. The data are referenced to Table 1; ^j^ Percentage of samples with three analytes contents outside the calculated limits.

## Supplementary Materials

Supplementary materials are available online. Supplementary file 1: cytotoxicity and effects of NO production of Ugonin J in lipopolysaccharides-stimulated RAW264.7 cells. Supplementary file 2: equations for calculation of the content limits of Quercetin and Ugonins. Supplementary file 3: the ^1^D-NMR spectroscopic data and the structure of Ugonin J.
