# Supplementary material for: Quality Control of the Root and Rhizome of Helminthostachys zeylanica (Daodi-Ugon) by HPLC Using Quercetin and Ugonins as Markers
Source: Molecules. 2017 Jul 5;22(7):1115. doi: 10.3390/molecules22071115 (PMC6152333; doi:10.3390/molecules22071115)
Supplement: Supplementary file 1 [file molecules-22-01115-s001.pdf]

molecules

MDPI

Supplementary File 1

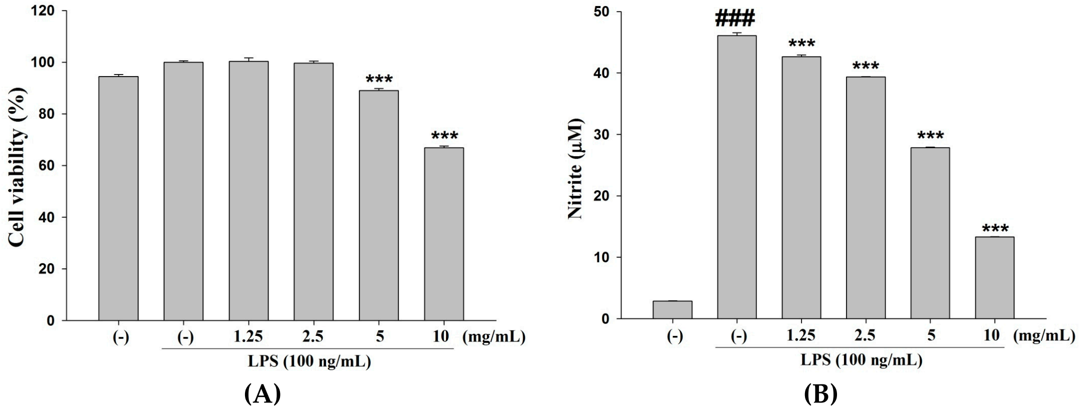

Figure S1. (A) Cytotoxicity and effects of (B) NO production of Ugonin J in lipopolysaccharides-stimulated RAW264.7 cells. Cells were pretreated with different concentrations of Ugonin J from 10, 5, 2.5, 1.25, or 0 μg/mL (0 referred to as (−)) for 1 h prior to the addition of 100 ng/mL LPS for 24 h. Cell viability assay was performed using MTT assay. Nitrite concentration in the medium was determined using Griess reagent. The data were presented as mean ± S.D. for the three different experiments performed in triplicate. ### $p < 0.001$ was compared with a sample of the control group (one-way ANOVA followed by Scheffe's multiple range tests). *** $p < 0.001$ was compared with the LPS-only group.

Supplementary File 2

Equations for calculation of the content limits of Quercetin and Ugonins

In this study, the content limits of Quercetin, Ugonin J, and Ugonin M were estimated with regard to the amount of analytes from twenty batches of *H. zeylanica* samples concerning the uncertainties caused by multiple factors which may induce variation on determination results.

Normally, for herbal quality analysis, those factors includes water content, standard purity, run-to-run precision and recovery. In general, the combined uncertainty was calculated with equation (1) by the square root of the total variance obtained from each of the uncertainty components, and the content limit was estimated with equation (2) (International Organization for the Standardization, 2008; Ellision and Williams, 2012; Barwick and Ellison, 2000):

$$u_c(C_a)/C_a = \sqrt{u(\mathrm{Pr})^2 + u(\mathrm{Rec})^2 + u(\mathrm{Pu})^2 + u(Cw)^2} \quad (1)$$

where C_a is the content of analyte in the sample (mg/kg); $u_c(C)$ is the combined standard uncertainty of contents of analyte in sample (mg/kg); $u(\mathrm{Pr}_a)$, $u(\mathrm{Rec}_a)$, $u(\mathrm{Pu}_a)$, and $u(Cw)$ are the standard uncertainty of the precision, recovery, purity and water content.

$$L = C - t_{(n-1)} \times SD/\sqrt{n} - REMU \times C \quad (2)$$

where L is the calculated content limit of analyte in the sampe; C is the average content of analyte in the samples (mg/kg); n = the number of samples (n=20); $t_{(n-1)}$ is the t value at 99% confidence level of significant, which is 2.539 for n=20; SD is the standard deviation of contents in samples with regard to the investigated analyte; REMU is the expended uncertainty which is 2-fold of the combined uncertainty calculated from equation (1).

Supplementary File 3

The structure of Ugonin J (yellow powder, $C_{25}H_{26}O_6$) was identified via a detailed analysis of ^{1}D-NMR spectroscopic data: ^{1}H NMR (Acetone-d6, 500 MHz) 13.26 (1H, s, 5-OH), 7.45 (1H, d, *J* = 1.6, H-2'), 7.41 (1H, dd, *J* = 1.6, 8.3, H-6'), 6.98 (1H, d, *J* = 8.3, H-5'), 6.54 (1H, s, H-3), 6.52 (1H, s, H-8), 4.42 (1H, d, *J* = 2.0, H-18), 4.25 (1H, d, *J* = 2.0, H-18), 2.97 (1H, br t, *J* = 12.4, H-9), 2.74 (1H, dd, *J* = 4.1, 12.4, H-9), 2.53 (1H, m, H-12), 2.43 (1H, dd, *J* = 4.1, 12.4, H-10), 1.92 (1H, m, H-12), 1.67 (1H, m, H-14), 1.58 (1H, m, H-13), 1.48 (1H, m, H-13), 1.25 (1H, m, H-14), 1.05 and 0.93 (each 3H, s, H-16, 17). These results were confirmed by comparison with previously published literature [1]. And the ^{1}D-NMR spectroscopic data of Ugonin M was shown in our previous report [2].

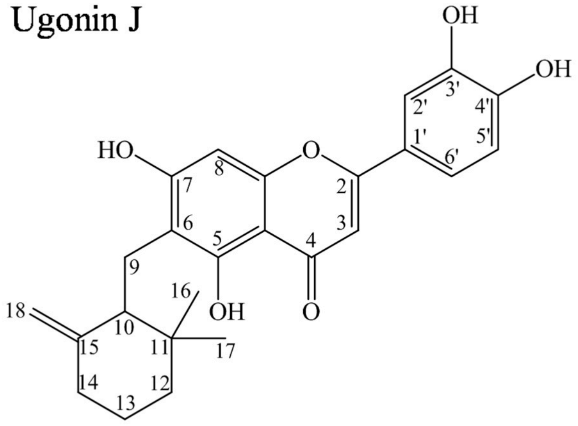

Figure S2. The structure of Ugonin J.

Reference

1. Huang Y.C.; Hwang T.L.; Chang C.S.; et al. Anti-inflammatory flavonoids from the rhizomes of *Helminthostachys zeylanica*. *Journal of Natural Products* 2009; **72**(7): 1273-8.
2. Wu KC, Huang SS, Kuo YH, et al. Ugonin M, a *Helminthostachys zeylanica* constituent, prevents LPS-induced acute lung injury through TLR4-mediated MAPK and NF-κB signaling pathways. *Molecules* 2017, 22, 573.
